# Cyclooxygenase-2 and β-Catenin as Potential Diagnostic and Prognostic Markers in Endometrial Cancer

**DOI:** 10.3389/fonc.2020.00056

**Published:** 2020-02-21

**Authors:** Lin Deng, Haiyan Liang, Yi Han

**Affiliations:** ^1^China-Japan Friendship Hospital, Beijing, China; ^2^Graduate School of Peking Union Medical College, Chinese Academy of Medical Sciences, Beijing, China; ^3^Beijing Haidian Center for Disease Control and Prevention, Beijing, China

**Keywords:** cyclooxygenase-2, β-catenin, wnt3a, endometrial cancer, prognostic marker, diagnostic marker

## Abstract

**Objectives:** Explore the diagnostic and prognostic value of cyclooxygenase-2 and wnt3a/β-catenin pathway in endometrial cancer.

**Methods:** A prospective cohort study of 93 women underwent hysterectomy at the China-Japan Friendship Hospital (61 patients with primary endometrial carcinoma, and 32 control patients with uterine prolapse or leiomyoma of uterus). Cox2 and β-catenin expression were determined by immunohistochemistry. The serum levels of cox2 and wnt3a were detected via ELISA assays.

**Results:** Patients with endometrial cancer showed overexpression of cox2 and β-catenin, as well as significantly higher serum levels of cox2 and wnt3a. The serum cox2 level, which is highly significant in predicting the risk of disease progression (RR, 9.617, 95% confidence interval, 1.162–79.622, *P* = 0.036), showed good diagnostic and prognostic potential, with cut-off of 55 U/L, but alongside β-catenin expression in tissues, were related to poor prognosis (RR, 12.426; 95% confidence interval, 1.618–95.450; *P* = 0.015).

**Conclusion:** Serum levels of cox2 and wnt3a exhibited diagnostic value for endometrial cancer. Cox2 serum levels and β-catenin expression also showed potential value of prognostic prediction. Cox2 serum levels might be a potential marker for early diagnosis and prognosis prediction in endometrial cancer.

## Introduction

Endometrial cancer (EC), accounting for 20–30%, is one of the three major female genital tract malignancies (1, 2). Most of them occurs in women aged 55–65 years, but was recently diagnosed in younger women (2). EC is divided into two types based on its dependence on estrogen, type I—estrogen-dependent cancer and type II—estrogen independent cancer (2, 3). Despite extensive research, the overall survival rate for EC patients is yet to improve, with 288,000 new cases diagnosed and 74,000 deaths registered each year worldwide (4). Therefore, identification of clinically prognostic risk factors is crucial to enhance the survival rate of EC patients.

Recently, some risk factors for EC such as age and genetics, were identified, but the reasons for the occurrence and progression of EC remain elusive (2). Recurrence and metastasis are important steps in the process of cancer progression (5). Several factors, including cyclooxygenase-2 (cox2) and β-catenin, are known to be abnormally regulated in EC (6, 7), suggesting their potential relevance to EC prognosis.

Cox2, which is involved in inflammatory processes, has been found to play a key role in prostaglandin biosynthesis (8, 9). Generally, the cox2 protein is only expressed in a limited number of cell types, whereas overexpressed in a variety of malignant tumors, suggesting its possible role in carcinogenesis (6, 10–15). Moreover, several mediatory factors, such as tumor promoters, proinflammatory cytokines and growth factors, have been reported to induce the upregulation of cox2 expression (16). Emerging data have demonstrated that cox2 is involved in many signaling pathways associated with cell proliferation, metastasis, and drug resistance (12, 13). Therefore, cox2 might be a potential factor affecting EC progression.

The β-catenin protein, an important intracellular transducer in wnt pathway, plays key roles in the maintenance of tissue homeostasis during adulthood (4, 17). Importantly, genetic aberrations have been identified in β-catenin expressed in EC tissue, suggesting that β-catenin might be associated with EC progression as well (7, 18, 19). The wnt/β-catenin pathway activates the expression of several important proteins responsible for the cell cycle, proliferation, and survival (20, 21). Moreover, β-catenin, together with E-cadherin, forms adherent junctions that mediate cell adhesion, which is a major factor affecting cell migration and invasion (22–24). Therefore, β-catenin might constitute a potential factor inducing EC growth and metastasis.

Despite these promising indications, the prognostic role and mechanisms of action of cox2 and β-catenin in EC have been widely disputed. Yet, to our knowledge, their impact on EC responses to chemotherapy and EC recurrence rates have never been evaluated. In this study, we applied immunohistochemical analysis and ELISA to compare the expression of cox2 and wnt pathway-related proteins in EC and normal endometrial tissues. We aimed to investigate the relationships of cox2 and β-catenin expression with various clinicopathological factors and then identify and delineate the potential mechanisms involved.

## Materials and Methods

### Patients

Patients were enrolled from December 2016 to December 2018 at the Department of Obstetrics and Gynecology, China-Japan Hospital in Beijing, China. In this prospective cohort study, we included 93 patients who underwent hysterectomy. Among these patients, 61 patients (age ranged from 41 to 78 years old) had primary EC and 32 patients (age ranged from 43 to 74 years old) had normal endometrium but underwent hysterectomy due to uterine prolapse or leiomyoma of the uterus. The study was approved by the Medical Ethics Committee of the China Japan Friendship Hospital. All participants gave their informed consent before being involved in the study. Histopathological results were evaluated by two independent gynecological pathologists (25). In each case, clinical information, such as age, presence of hypertension, BMI, and type 2 diabetes were collected, and image characteristics and pathological information were also obtained.

Inclusion criteria: complete clinical data; aged 40–80 years; histopathological diagnosis of endometrioid EC; without having received surgery, radiotherapy, chemotherapy or hormone therapy before enrollment; without carcinoma in other organs; without coagulation disorders; without endometriosis; without acute or chronic inflammation; without a history of taking non-steroidal anti-inflammatory drugs (NSAIDs); provision of a written consent form. Additionally, individuals with a history of endometrial polyp or endometrial atypical hyperplasia were excluded from the control group.

Serum samples obtained before surgery were stored at −80°C alongside collected tissue specimens and kept until molecular studies were performed. Tissue samples were obtained after the surgery and divided into pieces. Some were frozen at −80°C, and others were prepared for immunohistochemical (IHC) studies by fixation in 10% buffered formaldehyde solution for 48 h, routine dehydration, immersion in paraffin for embedding, and continuous slicing into 4-μm-thick sections according to standard procedures.

### Immunohistochemistry

Two biological markers (cox2 and β-catenin) were investigated via IHC study: cox2, β-catenin. Cox2 was assessed using monoclonal Ab (Rabbit Anti-cox2, ab15191, abcam, 1:200) and β-catenin was detected with a monoclonal Ab (Rabbit Anti-β-catenin, 8480, cst, 1:200). All the secondary antibodies and DAB stain were purchased from Beijing Zhongshan Golden Bridge Biotechnology Co., Ltd.

Deparaffinized sections by xylene and subsequently sequential immersion hydrated them by graded alcohol solutions (5). Antigen unmasking was performed in a container with Tris-EDTA buffer at PH 9.0, 0.5% Tween 20 using heat treatment in a microwave oven (800 W for 2 min without the sections and then turn to 150 W for 10 min with the sections). Sections were allowed to cool in the buffer for 30 min at 25°C. And then, rinsed them in phosphate buffer saline (PBS), 5 min each for three times. Next, incubated sections were treated with 3% hydrogen peroxide for 15 min at room temperature (25°C) to block the activity of endogenous peroxidase; this was followed by three washes with PBS and incubation with goat serum at 25°C for 40 min (5). Blocking reagent was removed, and sections were, then, probed with different monoclonal antibodies or with PBS (negative control) for 2 h at 25°C or overnight at 4°C. Sections were incubated with primary antibodies diluted with PBS and 1.5% bovine serum albumin (BSA). After primary antibody incubation, the sections were washed 3 times with PBS. Then, sections were incubated for 15 min with biotin-labeled secondary antibody. Washed with PBS for three times. Incubated sections with horseradish peroxidase (HRP)-labeled streptomycin working stain for 15 min, and washed 3 times with PBS (5). Finally, the sections were stained using diaminobenzidine as a chromogen and Meyers hematoxylin for counterstaining, then, mounted.

### Evaluation of Immunostaining

Immunostaining results were evaluated by two blinded pathologists using a light microscope, and a combined score based on the percentage of cells stained and staining intensity was used for assessment. Immunohistochemical cox2 and β-catenin staining were scored according to the criteria indicated by Krajewska et al. (26) and Kahane et al. (27). Immunohistochemically positive cells were considered as cells with membranes or cytoplasm containing yellow or brown granules. According to the percentage of immunohistochemically positive cells, the sections were graded as follows: 0, negative; 1, 1–25% positive cells; 2, 26–50% positive cells; 3, 51–75% positive cells; and 4, 76–100% positive cells (5). Immunostaining intensity was evaluated by the shade of brown granules and rated as follows: 0, negative; 1, weakly positive; 2, moderately positive; and 3, intensely positive (5). Both score sets were multiplied to derive composite points as follows: 0 point (–); 1–4 points (+); 5–8 points (++); and 9–12 points (+++). A combined staining score larger than or equal to 5 was considered as indicative of positive staining. The final score for each specimen was obtained by averaging the rating scores given by the two pathologists. If the scores given by both experts differed by more than 3 points, the evaluation would be repeated.

### ELISA Assay

ELISA was performed using a kit, according to the manufacturer's instructions. Firstly, set standard and experimental sample wells. Secondly, added 50 μl of standard solution to each standard wells and 50 μl combination (10 μl testing sample and 40 μl sample diluent) to the testing sample wells. Blank wells were left empty. Thirdly, added HRP-conjugated reagent 100 μl to each well and mixed. Next, covered the plate with an adhesive strip, and then, incubated at 37°C for 60 min. Washed the plate with washing solution for four times and then, inverted and blotted it on a clean paper (28). Subsequently, added chromogen solution A and B 50 μl in each well respectively, mixed, and incubated at 37°C in dark place for 15 min after mixing. Finally, added 50 μl stop solution to each well and measured optical density with microtiter plate reader at 450 nm within 15 min (29, 30).

### Statistical Analysis

Data were analyzed utilizing the SPSS 25.0 software and values were presented as average value ± standard deviation. Two groups' comparison: independent sample *t*-test or Mann–Whitney test. Comparisons of more than two groups: ANOVA analysis or non-parametric Kruskal-Wallis H tests. Chi-square test was used to compare rates (25). Receiver operating characteristic (ROC) curves using to evaluate the sensitivity and specificity of biomarkers in diagnosis. Spearman's and Pearson's correlation coefficients and logistic regression model were estimated to determine correlation (25). Kaplan-Meier and Cox regression methods were used to illustrate Progression Free Survival (PFS) and the relative risk (RR) for patients with normal biomarkers levels vs. elevated biomarkers levels. *P*-value < 0.05 was considered to have a statistically significance.

## Results

### Clinical Characteristics

The case group comprised 61 patients with EC, with a mean age of 58 years old (range from 41 to 78 years old), and a mean BMI of 24.28 kg/m^2^ (range from 18.61 to 30.12 kg/m^2^). The control group comprised 32 patients with leiomyoma of the uterus or uterine prolapse, with a mean age of 56 years (range from 43 to 74 years) and mean BMI of 24.62 kg/m^2^ (range from 18.22 to 30.12 kg/m^2^). There were no statistically significant differences in age distribution and BMI between the two groups (*P* > 0.05). No significant differences were observed in menopausal status, cesarean history, dysmenorrhea history, diabetes, and arterial hypertension as well (*P* > 0.05) (Table 1).

**Table 1:** Detailed clinical characteristics of the study participants.

	**Control**, ***n* = 32 (100%)**	**Case**, ***n* = 61 (100%)**	***P*-values**
Age			
Pre-menopausal	14 (43.75%)	23 (37.70%)	NS
Post-menopausal	18 (56.25%)	38 (62.30%)	
NA	0 (0%)	0 (0%)	
BMI			
≤ 24.9	17 (53.13%)	36 (59.02%)	NS
>24.9	15 (46.87%)	25 (40.98%)	
NA	0 (0%)	0 (0%)	
Diabetes			
Yes	0 (0%)	0 (0%)	NS
No	32 (100%)	61 (100%)	
NA	0 (0%)	0 (0%)	
Arterial hypertension			
Yes	4 (12.50%)	10 (16.39%)	NS
No	28 (87.50%)	51 (83.61%)	
NA	0 (0%)	0 (0%)	
Cesarean history			
Yes	6 (18.75%)	8 (13.11%)	NS
No	26 (81.25%)	53 (86.89%)	
NA	0 (0%)	0 (0%)	
Dysmenorrhea history			
Yes	3 (9.38%)	6 (9.84%)	NS
No	29 (90.62%)	55 (90.16%)	
NA	0 (0%)	0 (0%)	
Pregnancy history			
Yes	31 (96.87%)	49 (80.33%)	0.03
No	1 (3.13%)	12 (19.67%)	
NA	0 (0%)	0 (0%)	

*NA, not available; NS, not significant*.

Based on the classification by the Federation International of Gynecology and Obstetrics (FIGO), 26 (42.62%) patients had stage I, 19 (31.15%) had stage II, 14 (22.95%) had stage III, and 2 (3.28%) had stage IV EC. Among all patients, 38 (62.3%) had <1/2 myometrial invasion, and 23 (37.7%) had >1/2 myometrial invasion. Tumors were highly differentiated in 29 (47.54%) patients, moderately differentiated in 19 (31.15%) patients, and poorly differentiated in 13 (21.31%) patients. Ten (16.39%) patients had tumor-positive lymph nodes, whereas 51 (83.61%) patients had tumor-negative lymph nodes. Thirty-eight (62.3%) patients did not have vessel invasion, whereas 23 (37.7%) had (Table 2). The mean follow-up time was 24 ± 4.2 months (range from 13 to 33 months), and no patients died because of EC till now. However, 20 (32.8%) patients showed disease progression, such as recurrence, metastasis, or drug resistance (Table 2).

**Table 2:** Association of cox2, β-catenin of the 61 patients with endometrial cancer.

**Item**	***N***	**Cox2 positive**			**β-catenin positive**		
		***N***	***X*^**2**^**	***P***	***N***	***X*^**2**^**	***P***
FIGO stage			9.136	0.028		10.302	0.016
I	26	21			15		
II	19	19			17		
III	14	14			13		
IV	2	2			2		
Degree of differentiation			7.931	0.019		10.649	0.005
G1	29	24			18		
G2	19	19			16		
G3	13	13			13		
Myometrial invasion			5.000	0.082		8.787	0.012
<1/2	38	33			26		
≥1/2	9	9			7		
Full-thickness	14	14			14		
Lymph node metastasis			1.876	0.171		1.316	0.251
Negative	51	46			38		
Positive	10	10			9		
Vessel invasion			0.792	0.398		2.180	0.140
Negative	38	34			27		
Positive	23	22			20		

### Expression of Cox2 and β-Catenin in EC Tissues

**Figure 1:**
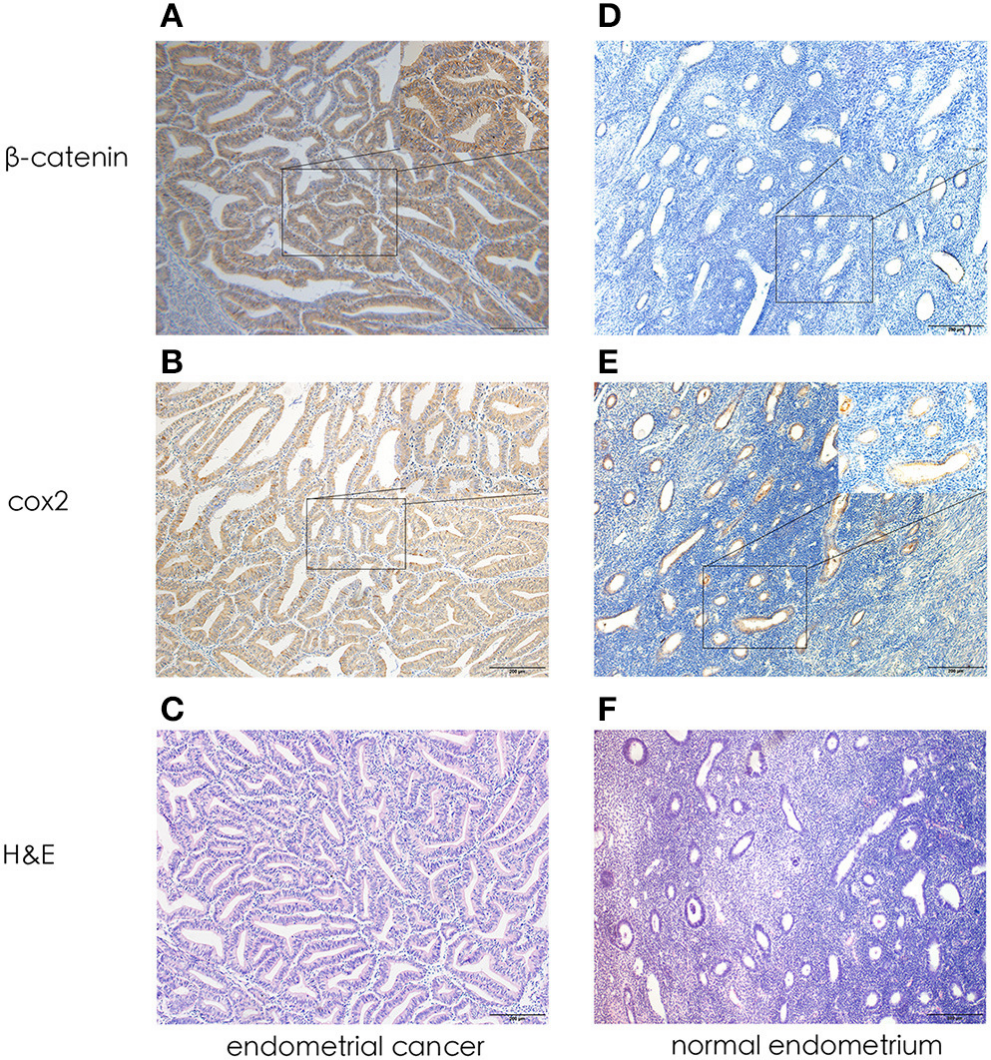
Representative photomicrographs of immunohistochemistry. **(A)** β-catenin expression in endometrial cancer (X 100, X400). **(B)** Cox2 expression in endometrial cancer (X 100, X400). **(C)** H&E in endometrial cancer (X 100). **(D)** β-catenin expression in normal endometrium (X 100, X400). **(E)** Cox2 expression in normal endometrium (X 100, X400). **(F)** H&E in normal endometrium (X 100).

In EC epithelial cells cytoplasm, intensely positive cox2 expression was observed: brown granules were diffused in the cytoplasm or linearly along the nucleus. Similarly, β-catenin was expressed in the cytoplasm or even, in the nucleus. Llittle or no immunoreactivity for cox2 and β-catenin were observed in normal endometrium. Cox2 and β-catenin positively expression were defined as tumor with a score of at least 5. Figure 1 shows a representative image of EC tissue with intensely immunoreactivity for both cox2 and β-catenin.

### Relationship Between Cox2 or β-Catenin Expression and Clinicopathological Parameters

Among 61 patients with EC, 55 (90.2%) exhibited a positive expression for cox2, whereas 47 (77%) patients showed positivity for expression for β-catenin. Protein expression of cox2 and β-catenin was associated with the FIGO stages, differentiation (*P* < 0.05) but not related to lymph node metastasis and vessel invasion (*P* > 0.05). β-catenin expression was also associated with myometrial invasion depth of tumors (*P* < 0.05). Differences in the expression of cox2 or β-catenin expression with clinicopathological parameters are shown in Table 2. Table S1 presents the Spearman rank correlation indices indicating a correlation between the expression of cox2 and β-catenin (*P* < 0.05).

### Relationship Between Cox2 or wnt3a Serum Levels and Clinicopathological Parameters

Serum levels of cox2 and wnt3a were significantly different between case and control patients. Serum levels of wnt3a was associated with the myometrial invasion depth of tumors and vessel invasion (*P* < 0.05). Serum level of cox2 was associated with the tumor differentiation (*P* < 0.05) changes in cox2 and wnt3a serum levels with clinicopathological parameters are shown in Table 3. Nonetheless, cox2 serum levels were positively correlated with wnt3a levels. Spearman rank correlation indices between cox2 and wnt3a serum levels were showed in Table S2.

**Table 3:** Association of cox2, wnt3a serum levels of the 61 patients with endometrial cancer.

**Item**	***N***	**Cox2** **>55 U/L**			**Wnt3a** **>25 ng/ml**		
		***N***	***X*^**2**^**	***P***	***N***	***X*^**2**^**	***P***
FIGO stage			6.034	0.110		1.074	0.783
I	26	21			21		
II	19	15			15		
III	14	14			12		
IV	2	2			2		
Degree of differentiation			6.206	0.045		5.959	0.051
G1	29	22			22		
G2	19	17			15		
G3	13	13			13		
Myometrial invasion			1.257	0.533		7.490	0.024
<1/2	38	31			28		
≥1/2	9	8			8		
Full-thickness	14	13			14		
Lymph node metastasis			3.515	0.061		0.587	0.443
Negative	51	42			41		
Positive	10	10			9		
Vessel invasion			1.150	0.283		5.542	0.019
Negative	38	31			28		
Positive	23	21			22		

### Expression of Cox2 and β-Catenin in Tissues Is Associated With Patient Diagnosis and Progression-Free Survival (PFS) Time

**Figure 2:**
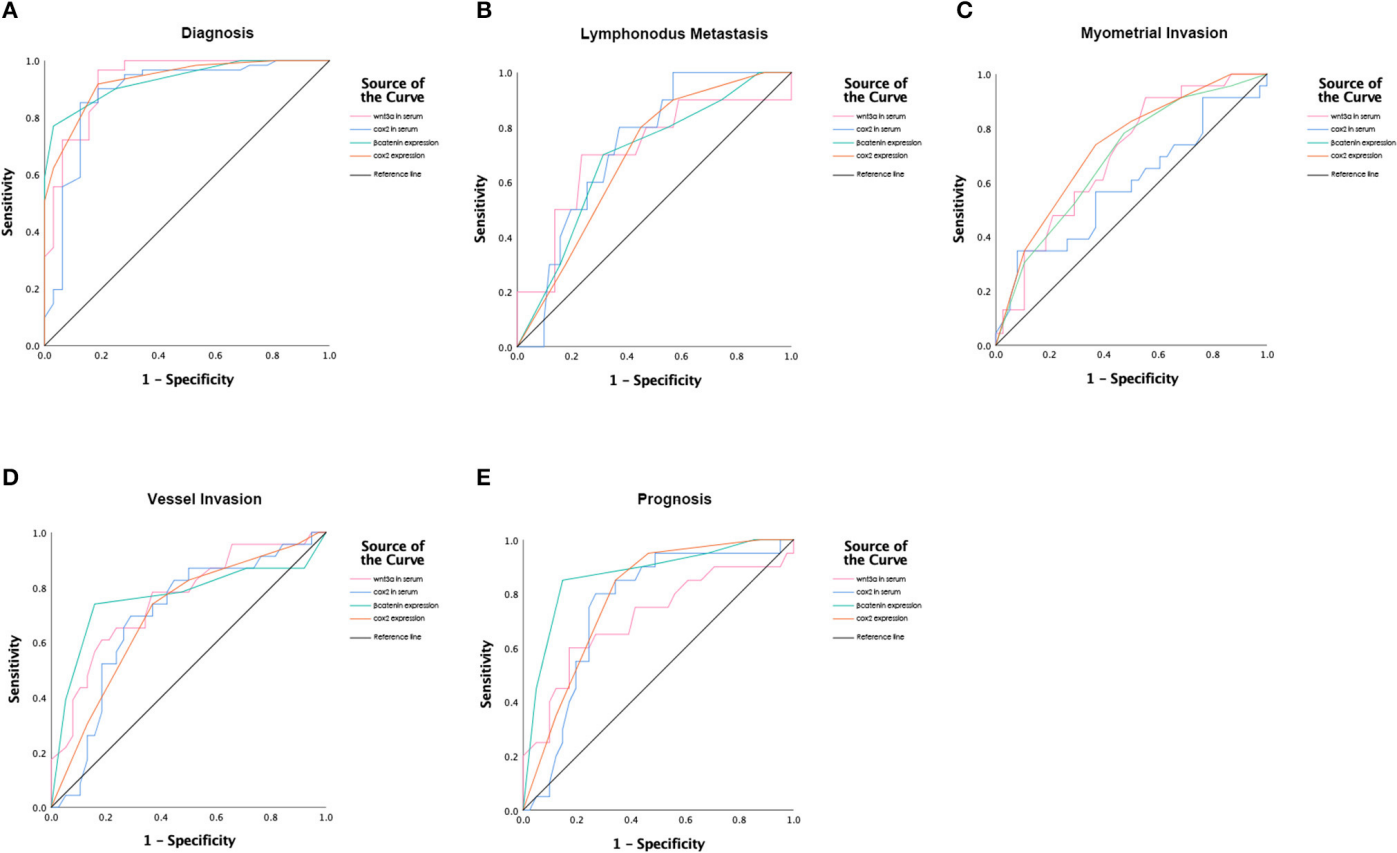
Receiver operating characteristic (ROC) curves for **(A)** Diagnosis; **(B)** Prognostic values of lymph node invasion; **(C)** Prognostic values of myometrial invasion; **(D)** Prognostic values of vessel invasion; **(E)** Poor prognostic values.

Tissue expression of cox2 and β-catenin indicated their prognostic potential in terms of vessel invasion, the depth of myometrial invasion and disease progress. Expression of cox2 differed significantly when patients were stratified according to vessel invasion, myometrial invasion status, and prognosis status, each with an area under the curve (AUC) of 0.702 (sensitivity of 73.9% and specificity of 63.2%), 0.732 (sensitivity of 82.6% and specificity of 50%), 0.789 (sensitivity of 85% and specificity of 65.9%), respectively. For β-Catenin, the AUC was 0.763 (sensitivity of 78.3% and specificity of 52.6%) for vessel invasion, 0.692 (sensitivity of 78.3% and specificity of 52.6%) for depth of myometrial invasion, and 0.869 (sensitivity of 85% and specificity of 85.4%) for prognosis status. The ROC curves according to lymph node metastasis did not have statistically significance for cox2 and β-Catenin expression. Receiver operating characteristics (ROC) curves were shown in Figure 2 and the index of ROC curves are showed in Table S3.

**Figure 3:**
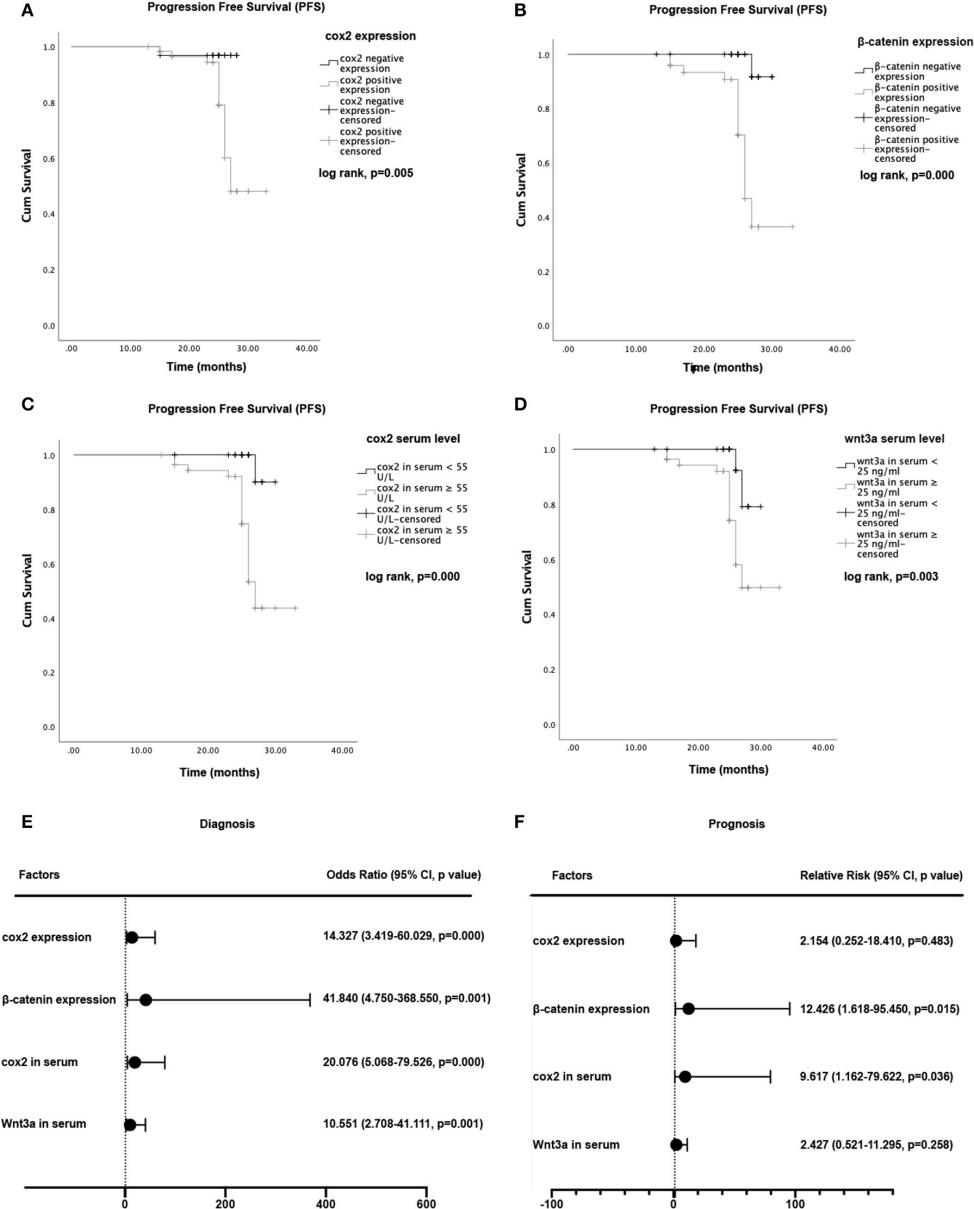
Kaplan-Meier curves for progression-free survival (PFS) and hazard ratios for tumor progression. **(A)** Comparison of PFS between cox2 positive group and the negative group. **(B)** Comparison of PFS between β-catenin positive group and negative group. **(C)** Comparison of PFS in endometrial cancer patients between those with cox2 serum levels <55 U/L and those with cox2 serum levels ≥ 55 U/L. **(D)** PFS in patients with wnt3a serum levels <25 ng/ml vs patients with wnt3a serum levels ≥ 25 ng/ml. **(E)** Odds ratio (OR) for diagnosis. **(F)** Relative Risk (RR) for disease progression. For diagnosis and prognosis prediction, cox2 serum level and β-catenin expression demonstrated a predictive ability.

Logistic regression models were constructed to predict the relationship between EC diagnosis and the expression of cox2 and β-catenin. Regarding disease diagnosis, our logistic model showed an odds ratios (OR) of 41.840 (95% confidence interval, 4.750–368.550, *P* = 0.001) for β-Catenin, and 14.327 (95% confidence interval, 3.419–60.029, *P* = 0) for cox2. Results from Kaplan-Meier survival analysis showed that both cox2 and β-catenin expression were significantly associated with patients' PFS, which means the survival time without recurrence, death, drug-resistance or metastasis. Take β-catenin and cox2 together in the Cox proportional hazard model, the expression of β-catenin had statistically significance on patients' prognosis (β-Catenin: RR, 12.426; 95% confidence interval, 1.618–95.450; *P* = 0.015), suggesting its potential as a single risk factor to EC prognosis (Figure 3).

### Serum Level of Cox2 Might Be Good Diagnostic and Prognostic Factor for EC

Serum levels of cox2 and wnt3a showed the prediction potential in EC diagnosis (cox2: AUC of 0.887, sensitivity of 95.1% and specificity of 71.9%, *P* = 0; wnt3a: AUC of 0.931, sensitivity of 96.7% and specificity of 81.2%, *P* = 0). Serum levels of cox2 and wnt3a also showed the prognostic potential in vessel invasion (cox2: AUC of 0.696, sensitivity of 73.9% and specificity of 63.2%, *P* = 0.011; wnt3a: AUC of 0.757, sensitivity of 78.3% and specificity of 63.2%, *P* = 0.001) and lymph node metastasis (cox2: AUC of 0.732, sensitivity of 80% and specificity of 62.7%, *P* = 0.021; wnt3a: AUC of 0.711, sensitivity of 70% and specificity of 76.5%, *P* = 0.036) and poor prognosis (cox2: AUC of 0.752, sensitivity of 80% and specificity of 73.2%, *P* = 0.002; wnt3a: AUC of 0.711, sensitivity of 75% and specificity of 58.5%, *P* = 0.008). ROC curves were shown in Figure 2 and the index of ROC curves are shown in Table S3.

Logistic regression models were built to predict the relationship between serum levels of cox2 or wnt3a and EC diagnosis. The logistic regression model showed an OR of 10.551 (95% confidence interval, 2.708–41.111, *P* = 0.001) for wnt3a, and 20.076 (95% confidence interval, 5.068–79.526, *P* = 0.000) for cox2. The cox2 and wnt3a serum levels also showed meaningful influence on the PFS of EC patients. Results obtained from Kaplan-Meier survival analysis showed that both cox2 and wnt3a serum levels were significantly associated with patient survival (*P* < 0.05). Take wnt3a and cox2 levels together in the Cox proportional hazard model with cut-off values of 55 U/L for cox2 and 25 ng/ml for wnt3a. Serum levels of cox2 showed a statistically significant influence on PFS and suggested as a single risk factor (cox2: RR, 9.617, 95% confidence interval, 1.162–79.622, *P* = 0.036) (Figure 3).

## Discussion

Curettage and pathohistological examinations are still regarded as the gold standard for EC diagnosis due to the absence of non-invasive biomarkers for predicting the presence and extent of the disease (25). Surgery and chemotherapy or radiotherapy after surgery remain the major treatment options for EC. Recurrence and metastasis are the major causes of EC patient death. Thus, early detection using diagnostic and prognostic biomarkers, thus, has important clinical implications as these could help screen asymptomatic women and distinguish patients with a high risk of disease progression from those with better prognosis. However, the number of studies on the exploration of biomarkers that could help early diagnosis and predict the prognosis of patients with EC is still limited. Although there have been several studies in EC patients examining serum indices, such as cancer antigen 125 (CA125), human epididymis protein (HE4), and VEGF (25, 31–33), their reliability and repeatability still lack consensus.

In recent years, several studies have explored the relationship between cox2 expression and patients' prognosis in EC. However, to our knowledge, none of them have evaluated the serum levels of cox2 in patients with EC. The current study is the first to detect and measure serum levels of cox2 and wnt3a in EC patients using ELISA, and is the first to show that IHC expression and serum levels of biomarkers correlated with the clinical outcome of EC (34). In this cohort study, we examined cox2 and wnt3a serum levels using ELISA and tissue expression using IHC. The results showed that cox2 and wnt3a serum concentrations correlated with diagnosis, prognosis, and disease extent factors. Similarly, immunochemical analysis revealed that overexpression of cox2 and β-catenin is associated with EC prognosis. Subsequently, Cox regression models showed that cox2 serum level and β-catenin expression were single risk factor to EC prognosis, suggesting that the cox2 serum level might be used as a potential biomarker for early non-invasive diagnosis and further prognosis prediction of EC. Additionally, β-catenin expression might be another assistant marker for further prognosis prediction.

Cox2 expression in normal tissues is limited; however, when stimulated by cytokines, growth factors, and oncogenes, its expression is upregulated (16). Notably, cox2 is known to play a key role in the development of various tumor types (9–12, 14). Previous studies have indicated that cox2 could participate in the processes of cell proliferation, migration, and invasion, thus playing a key role in tumor growth and metastasis (12, 14, 15). However, studies investigating the relationship between cox2 and EC are still limited, and cox2 serum levels in patients with EC have never been reported. In the present study, we could not detect or only detected very low cox2 expression in normal endometrial tissues, whereas in EC tissues, 90.2% positive cox2 expression was reported. Patients with cox2 overexpression were more likely to have poor prognosis. Moreover, overexpression of cox2 also showed relationships with factors related to EC progression, such as FIGO stage, differentiation and myometrial invasion (*P* < 0.05 for all), which were similarly to previous studies (6, 10, 34). Additionally, we evaluated the cox2 serum levels and observed that they were significantly elevated in patients with EC compared to those with normal endometrium. The present results also indicated that cox2 serum levels can help differentiate among patients with poorly differentiation and prognosis, suggesting its potential prognostic value. Importantly, our study showed that cox2 serum levels with a cut-off value of 55 U/L were a better predictor of early EC diagnosis, depth of myometrial invasion, lymph node involvement, vessel invasion, and further prognosis. Kaplan-Meier analysis and Cox regression indicated that patients with higher serum levels of cox2 were more likely to have poor prognosis, such as recurrence, metastasis, and drug-resistance. Cox regression model also indicated cox2 serum level as a single risk factor for EC patients (RR, 9.617, 95% confidence interval, 1.162–79.622, *P* = 0.036), and was more specific and sensitive compared to the cox2 tissue expression (RR, 2.154; 95% confidence interval, 0.252–18.410, *P* = 0.483), indicating that cox2 serum levels might potentially be a more efficient marker for early diagnosis and prognosis prediction in high risk EC patients.

Wnt signaling, a cellular pathway pivotal for embryonic development and tissue homeostasis, was found to be effective in primary axis formation, organogenesis, stem cell proliferation, and cell fate decisions as well (35–40). Its aberrant activation has been associated with the majority of human malignancies, including EC (41, 42). In particular, β-catenin is the primary mediator of the oncogenic effect in this signaling pathway (17, 21). Almost 30–85% of type I EC cases show nuclear accumulation of β-catenin in the epithelium (43–45). Thus, it is important to understand the role of wnt/β-catenin signaling in EC. A recent study reported that wnt/β-catenin pathway dysregulation to be an important factor in young EC patients with poor prognosis (45–47). Genetic mutations of the wnt/β-catenin signaling pathway members are mostly detected in patients with more aggressive histologic type of EC and are related to worse prognosis and poor overall survival rate (4, 10, 45). These findings are similar to the results of our study. In this study, we detected the wnt3a serum levels and β-catenin tissue expression, and found that high wnt3a serum levels and β-catenin overexpression were correlated with poor prognosis. Importantly, our study showed that serum levels of wnt3a with a cut-off value of 25 ng/ml were a good predictor for EC early diagnosis. Positive β-catenin expression was important in predicting prognosis. Kaplan-Meier analysis indicated that patients with higher serum levels of wnt3a and β-catenin expression were more likely to have a poor prognosis, supporting that the wnt/β-catenin pathway is important for predicting EC prognosis. But the cox regression model showed that the RR value of wnt3a (RR, 2.427; 95% confidence interval, 0.521–11.295, *P* = 0.258) was not statically significant, and the OR value (10.551, 95% confidence interval, 2.708–41.111, *P* = 0.001) was lower than that of cox2 serum levels. Therefore, we speculated that cox2 might regulate EC growth and metastasis via the wnt/β-catenin pathway and could function as a potential predictive biomarker for EC diagnosis and prognosis. Detecting cox2 serum levels might thus conveniently and efficiently aid early diagnosis of EC and prognosis prediction. As β-catenin expression was also a risk factor for prognosis, combined the serum levels of cox2 with β-catenin expression, the predictive value could be enhanced.

This study had several limitations as well. Firstly, due to the currently limited experimental technology, we only detected the expression levels of cox2 and wnt pathway-related proteins using immunochemistry and ELISA assays and assessed the correlation between these biomarkers and the diagnosis and prognosis of EC based on these results. However, the detailed molecular regulatory mechanisms of the action of these proteins in EC were not delineated or verified through *in vitro* or *in vivo* experiments; these aspects require further investigation. What's more, this study only included the Asiatic population and endometrioid histology, which may cause some limitation in generalizing the conclusion. Additionally, in this study, we included some young patients, which might have some genetic influence and bias. So, the age range scope could be reduced and microsatellite instability related proteins should be detected to excluded individuals with systemic predisposition to cancer like lynch syndrome. In addition, as this is an ongoing prospective study, the sample size and follow up time have been limited up to now. The sensitivity was high but specificity was lower, moreover, the overall survival time for patients did not reveal a significant difference, the reliability and validity of the cut-off value were not evaluated as well. Further big sample size, multi-center, randomly clinical controlled trials are still needed.

## Conclusion

In conclusion, our study showed that cox2 and β-catenin were overexpressed in EC tissues and the serum levels of cox2 and wnt3a in patients with EC were elevated as well. Cox2 serum level and β-catenin expression were positively correlated with EC diagnosis and poor prognosis. Cox2 and β-catenin might potentially function as a predictable biomarkers for EC patients. This study had certain limitations, but provides a basis for further exploration into the mechanisms of action of cox2 in EC and its potential clinical application as a diagnostic and prognostic marker.

## Data Availability Statement

The datasets used and analyzed during the current study are available from the corresponding author on reasonable request.

## Ethics Statement

The studies involving human participants were reviewed and approved by the Medical Ethics Committee of the China Japan Friendship Hospital. The patients/participants provided their written informed consent to participate in this study.

## Author Contributions

LD carried out the experiments and wrote the manuscript. All authors analyzed the experimental results and revised the manuscript.

### Conflict of Interest

The authors declare that the research was conducted in the absence of any commercial or financial relationships that could be construed as a potential conflict of interest.

## Abbreviations

cox2: cyclooxygenase 2
wnt3a: Wnt family member 3A
EC: endometrial cancer
ROC: receiver operating characteristic
PFS: progression free survival
RR: Relative Risk
OR: Odds ratio
AUC: area under the curve
CA125: cancer antigen 125
HE4: human epididymis protein
IHC: immunohistochemistry
NSAIDs: non-steroidal anti-inflammatory drugs
HRP: horseradish peroxidase
H&E: hematoxylin-eosin staining.
