# Corrigendum: Cyclooxygenase-2 and β-Catenin as Potential Diagnostic and Prognostic Markers in Endometrial Cancer

**DOI:** 10.3389/fonc.2020.582704

**Published:** 2020-11-03

**Authors:** Lin Deng, Haiyan Liang, Yi Han

**Affiliations:** ^1^China-Japan Friendship Hospital, Beijing, China; ^2^Graduate School of Peking Union Medical College, Chinese Academy of Medical Sciences, Beijing, China; ^3^Beijing Haidian Center for Disease Control and Prevention, Beijing, China

**Keywords:** cyclooxygenase-2, β-catenin, wnt3a, endometrial cancer, prognostic marker, diagnostic marker

In the original article, there was a mistake in the Supplementary Table named ***Table S3 AUCs, Sensitivity, Specificity of cox2, β-catenin and wnt3a in predicting diagnosis, myometrial invasion, vessel invasion, lymph node metastasis, and poor prognosis*** as published. The specificity of wnt3a in serum for myometrial invasion is 0.316 and the p value is 0.010. The corrected Supplementary Table S3 appears below.

**Table S3:** AUCs, Sensitivity, Specificity of cox2, β-catenin and wnt3a in predicting diagnosis, myometrial invasion, vessel invasion, lymph node metastasis, and poor prognosis.

	**Item**	**AUCs**	**Sensitivity**	**Specificity**	**95% CI**	***P***
Diagnosis	Cox2 expression	0.931	0.918	0.812	0.882–0.981	0
	β-catenin expression	0.933	0.902	0.750	0.886–0.980	0
	Cox2 in serum	0.887	0.951	0.719	0.805–0.968	0
	Wnt3a in serum	0.931	0.967	0.812	0.873–0.981	0
Myometrial invasion	Cox2 expression	0.732	0.826	0.500	0.605–0.860	0.003
	β-catenin expression	0.692	0.783	0.526	0.556–0.6827	0.013
	Cox2 in serum	0.590	0.913	0.237	0.437–0.744	0.240
	Wnt3a in serum	0.698	0.913	0.316	0.566–0.830	0.010
Vessel invasion	Cox2 expression	0.702	0.739	0.632	0.567–0.837	0.009
	β-catenin expression	0.763	0.783	0.526	0.619–0.906	0.001
	Cox2 in serum	0.696	0.739	0.632	0.558–0.834	0.011
	Wnt3a in serum	0.757	0.783	0.632	0.631–0.884	0.001
Lymph node metastasis	Cox2 expression	0.692	0.800	0.549	0.537–0.847	0.056
	β-catenin expression	0.693	0.700	0.686	0.522–0.864	0.055
	Cox2 in serum	0.732	0.800	0.627	0.596–0.869	0.021
	Wnt3a in serum	0.711	0.700	0.765	0.517–0.905	0.036
Prognosis	Cox2 expression	0.789	0.850	0.659	0.676–0.902	0
	β-catenin expression	0.869	0.850	0.854	0.767–0.971	0
	Cox2 in serum	0.752	0.800	0.732	0.623–0.881	0.002
	Wnt3a in serum	0.711	0.750	0.585	0.561–0.861	0.008

An author was incorrectly labeled as **Haiyan Liang**^**1**^, as she is the co-correspondence author. The correct label is Haiyan Liang^1*^.

The authors apologize for these errors and state that this does not change the scientific conclusions of the article in any way. The original article has been updated.

