# Supplementary material for: Cyclooxygenase-2 and β-Catenin as Potential Diagnostic and Prognostic Markers in Endometrial Cancer
Source: Front Oncol. 2020 Feb 21;10:56. doi: 10.3389/fonc.2020.00056 (PMC7046792; doi:10.3389/fonc.2020.00056)
Supplement: Supplementary file 1 [file Table_1.docx]

Table S1 Correlation of cox2 expression with β-catenin expression of these 93 patients

|  |  | β-catenin | | | | |
| --- | --- | --- | --- | --- | --- | --- |
|  |  | Positive | Negative | *X*^2^ | *P* | r_s_ |
| Cox2 | Positive | 46 | 16 | 37.975 | 0.0 | 0.639* |
|  | Negative | 2 | 29 |  |  |  |

*：P<0.01
