# Supplementary material for: Cyclooxygenase-2 and β-Catenin as Potential Diagnostic and Prognostic Markers in Endometrial Cancer
Source: Front Oncol. 2020 Feb 21;10:56. doi: 10.3389/fonc.2020.00056 (PMC7046792; doi:10.3389/fonc.2020.00056)
Supplement: Supplementary file 2 [file Table_2.docx]

Table S2 Correlation of cox2 levels with wnt3a levels in serum of these 93 patients

|  |  | Wnt3a | | | | |
| --- | --- | --- | --- | --- | --- | --- |
|  |  | <25 ng/ml | ≥25 ng/ml | *X*^2^ | *P* | r_s_ |
| Cox2 | <55 U/L | 28 | 9 | 30.822 | 0.0 | 0.576* |
|  | ≥55 U/L | 10 | 46 |  |  |  |

*：P<0.01
