# Supplementary material for: Cyclooxygenase-2 and β-Catenin as Potential Diagnostic and Prognostic Markers in Endometrial Cancer
Source: Front Oncol. 2020 Feb 21;10:56. doi: 10.3389/fonc.2020.00056 (PMC7046792; doi:10.3389/fonc.2020.00056)
Supplement: Supplementary file 3 [file Table_3.DOCX]

Table S3 AUCs, Sensitivity, Specificity of cox2, β-catenin and wnt3a in predicting diagnosis, myometrial invasion, vessel invasion, lymph node metastasis, and poor prognosis

| Item | | AUCs | Sensitivity | Specificity | 95% CI | P |
| --- | --- | --- | --- | --- | --- | --- |
| Diagnosis | Cox2 expression | 0.931 | 0.918 | 0.812 | 0.882-0.981 | 0 |
|  | β-catenin expression | 0.933 | 0.902 | 0.750 | 0.886-0.980 | 0 |
|  | Cox2 in serum | 0.887 | 0.951 | 0.719 | 0.805-0.968 | 0 |
|  | Wnt3a in serum | 0.931 | 0.967 | 0.812 | 0.873-0.981 | 0 |
| Myometrial invasion | Cox2 expression | 0.732 | 0.826 | 0.500 | 0.605-0.860 | 0.003 |
|  | β-catenin expression | 0.692 | 0.783 | 0.526 | 0.556-0.6827 | 0.013 |
|  | Cox2 in serum | 0.590 | 0.913 | 0.237 | 0.437-0.744 | 0.240 |
|  | Wnt3a in serum | 0.698 | 0.913 | 0.316 | 0.566-0.830 | 0.010 |
| Vessel invasion | Cox2 expression | 0.702 | 0.739 | 0.632 | 0.567-0.837 | 0.009 |
|  | β-catenin expression | 0.763 | 0.783 | 0.526 | 0.619-0.906 | 0.001 |
|  | Cox2 in serum | 0.696 | 0.739 | 0.632 | 0.558-0.834 | 0.011 |
|  | Wnt3a in serum | 0.757 | 0.783 | 0.632 | 0.631-0.884 | 0.001 |
| Lymph node metastasis | Cox2 expression | 0.692 | 0.800 | 0.549 | 0.537-0.847 | 0.056 |
|  | β-catenin expression | 0.693 | 0.700 | 0.686 | 0.522-0.864 | 0.055 |
|  | Cox2 in serum | 0.732 | 0.800 | 0.627 | 0.596-0.869 | 0.021 |
|  | Wnt3a in serum | 0.711 | 0.700 | 0.765 | 0.517-0.905 | 0.036 |
| Prognosis | Cox2 expression | 0.789 | 0.850 | 0.659 | 0.676-0.902 | 0 |
|  | β-catenin expression | 0.869 | 0.850 | 0.854 | 0.767-0.971 | 0 |
|  | Cox2 in serum | 0.752 | 0.800 | 0.732 | 0.623-0.881 | 0.002 |
|  | Wnt3a in serum | 0.711 | 0.750 | 0.585 | 0.561-0.861 | 0.008 |
